# Bias Detection: Study Identifies Instruments for Evaluating Animal Studies

**DOI:** 10.1289/ehp.121-a285

**Published:** 2013-09-01

**Authors:** Kellyn S. Betts

**Affiliations:** Kellyn S. Betts writes about environmental contaminants, hazards, and technology for solving environmental problems for publications including *EHP* and *Environmental Science & Technology*.

No good scientist wants to produce, or be accused of producing, a poorly conducted study. Even so, toxicologists conducting animal studies are not widely expected to document the steps they take to ensure internal validity—that is, to prevent their results from being skewed due to a methodological issue (a concept known as risk of bias). This is likely to change soon, and a team of researchers at the University of California, San Francisco (UCSF) has taken a first step toward a solution by conducting a systematic literature review that identifies 30 instruments for evaluating risk of bias in animal research.^1^

“Risk of bias” refers specifically to the introduction of systematic errors as a result of the way a study was conducted; it is unrelated to prejudice or manipulation of results to achieve a desired outcome. Other aspects of study quality include external validity (the extent to which results can be generalized) and reporting quality (descriptions of the experiment design, conduct, and analysis).^2^ Tools to assess these other aspects of study quality are familiar to toxicologists,^3^^,^^4^^,^^5^ but the concept of risk of bias is relatively new in environmental health, says Kris Thayer, director of the National Toxicology Program’s Office of Health Assessment and Translation (OHAT), who was not involved in the new review.

Thayer’s group is leading efforts to incorporate systematic review methodology into OHAT evaluations.^6^ She says it’s important, when conducting these evaluations, to clearly define which aspects of study quality are being considered and to present them as discrete elements. She lauds the new review for providing a starting point for determining how to evaluate the risk of bias in animal studies.

The review presents an inventory of different approaches for assessing existing study-quality tools and summarizes what is known about specific factors as sources of systematic bias. “Although there is a well-developed and empirically based literature on how to evaluate the risk of bias of randomized controlled clinical trials, less is known about how to do this for animal studies,” wrote the team of UCSF researchers, who were led by Lisa Bero, an expert in systematic review methods and evidence-based health care.

Bero and her coauthors combed through 45 years of MEDLINE publications and found 3,731 articles describing instruments for assessing risk of bias in animal studies. They identified 30 instruments that assess anywhere from 2 to 25 criteria associated with the risk of bias, methodological, or reporting criteria.

Rather than pinpointing a single “best instrument,” the authors attempt to identify the core set of procedures important for evaluating risk of bias in animal studies, Bero says. These procedures include some process of random allocation to experimental groups; a blinded assessment of outcome; statistical tools to be sure that studies aren’t underpowered; recording both inclusion and exclusion criteria to show that the animals were appropriate for the study; and ensuring that all collected data were reported.

**Figure 1:**
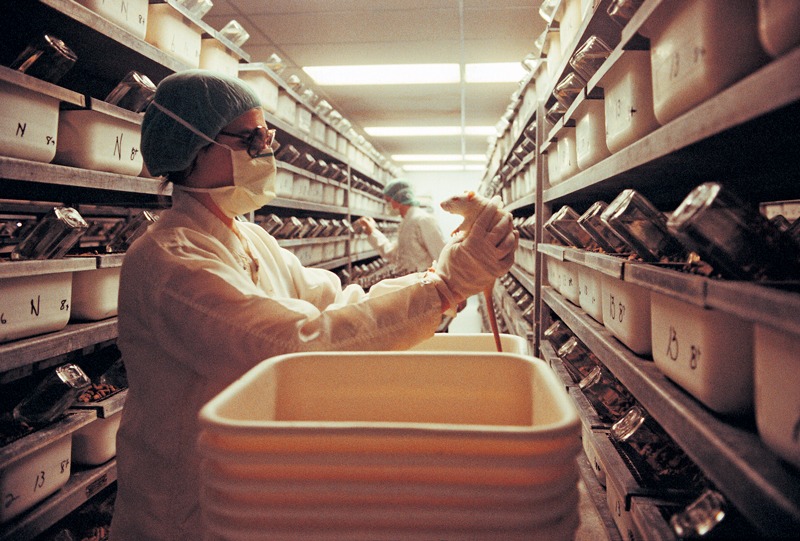
Bias, or the introduction of errors into experiments, may be one explanation for equivocal research findings. © Getty Images

The authors acknowledge that they may not have identified all published assessment instruments because they searched only for English-language publications using just one database. “However,” they wrote, “both our consultation with a librarian and the large pool of studies identified through the electronic search suggests that it was comprehensive.”^1^

Study coauthor Tracey Woodruff of UCSF’s Department of Obstetrics, Gynecology and Reproductive Sciences and Program on Reproductive Health and the Environment, calls the review “a foundational piece of a broader method that is going to greatly improve our ability to evaluate environmental chemicals.” Woodruff’s group is working to establish how the systematic reviews initially developed for use in the decades-old field of evidence-based medicine can be applied to environmental health and has developed a strategy known as the Navigation Guide.^7^ Woodruff’s overarching goal is to encourage medical practitioners to educate their patients about hazardous environmental exposures, particularly ones that may affect their reproductive health.^8^

Malcolm R. Macleod, a professor at the University of Edinburgh’s Centre for Clinical Brain Sciences, who was not involved in the study, calls the review a small but “really important” contribution toward reducing the risk of bias, says. Macleod’s interest in the subject sprang from his work on clinical trials in which drug candidates recommended by animal stroke studies ended up failing in humans. His subsequent analysis^9^ demonstrated how study quality and publication bias in those animal studies led to a major overstatement of the drugs’ efficacy.

His publications and others document that lack of randomization or blinding, failure to specify inclusion/exclusion criteria or use of comorbid animals, and lack of statistical power in animal studies have inflated the estimates of the effects of pharmaceutical interventions for a number of human maladies.^10^^,^^11^^,^^12^^,^^13^^,^^14^ They inspired high-profile pleas to establish better reporting criteria that incorporates risk-of-bias assessments.^15^^,^^16^

## References

[1] Krauth D Instruments for assessing risk of bias and other methodological criteria of published animal studies: a systematic review. Environ Health Perspect 121 9 985 992 2013; http//dx..org/10.1289/ehp.1206389. 23771496 PMC3764080

[2] Higgins JPT, Green S, eds. Cochrane Handbook for Systematic Reviews of Interventions. West Sussex, UK:John Wiley & Sons Ltd. (2008).

[3] Hooijmans CR (2010). A gold standard publication checklist to improve the quality of animal studies, to fully integrate the Three Rs, and to make systematic reviews more feasible.. Altern Laboratory Anim.

[4] Kilkenny C Animal research: reporting *in vivo* experiments: the ARRIVE guidelines. Br J Pharmacol 160 7 1577 1579 2010; http//dx..org/10.1111/j.1476-5381.2010.00872.x. 20649561 PMC2936830

[5] Schneider K “ToxRTool”, a new tool to assess the reliability of toxicological data. Toxicol Lett 189 2 138 144 2009; http//dx..org/10.1016/j.toxlet.2009.05.013. 19477248

[6] NTP. OHAT Implementation of Systematic Review [website]. Research Triangle Park, NC:National Toxicology Program, Department of Health and Human Services (updated 6 August 2013). Available: http://goo.gl/5gtvxJ [accessed 8 August 2013].

[7] Clinical Practice & Policy: Navigation Guide Strategy [website]. Oakland, CA:University of California San Francisco Program on Reproductive Health and the Environment (updated 6 February 2013). Available: http://prhe.ucsf.edu/prhe/navigationguide_strategy.html [accessed 8 August 2013].

[8] Woodruff TJ An evidence-based medicine methodology to bridge the gap between clinical and environmental health sciences. Health Aff 30 5 931 937 2011; http//dx..org/10.1377/hlthaff.2010.1219. PMC6663095 21555477

[9] Sena ES Publication bias in reports of animal stroke studies leads to major overstatement of efficacy. PLoS Biol 8 3 e1000344 2010; http//dx..org/10.1371/journal.pbio.1000344. 20361022 PMC2846857

[10] Vesterinen H Improving the translational hit of experimental treatments in multiple sclerosis. Mult Scler 16 9 1044 1055 2010; http//dx..org/10.1177/1352458510379612. 20685763

[11] Rooke EDM Dopamine agonists in animal models of Parkinson’s disease: a systematic review and meta-analysis. Parkinsonism Relat Disord 17 5 313 320 2011; http//dx..org/10.1016/j.parkreldis.2011.02.010. 21376651

[12] Bebarta V Emergency medicine animal research: does use of randomization and blinding affect the results? Acad Emerg Med 10 6 684 687 2003; http//dx..org/10.1111/j.1553-2712.2003.tb00056.x. 12782533

[13] Hirst TC Systematic review and meta-analysis of temozolomide in animal models of glioma: was clinical efficacy predicted? Br J Cancer 108 1 64 71 2013; http//dx..org/10.1038/bjc.2012.504. 23321511 PMC3553514

[14] Currie GL Animal models of bone cancer pain: systematic review and meta-analyses. Pain 154 6 917 926 2013; http//dx..org/10.1016/j.pain.2013.02.033. 23582155

[15] National Research Council Institute for Laboratory Animal Research. Guidance for the Description of Animal Research in Scientific Publications. Washington DC:The National Academies Press (2011). 22379656

[16] Landis SC A call for transparent reporting to optimize the predictive value of preclinical research. Nature 490 7419 187 191 2012; http//dx..org/10.1038/nature11556. 23060188 PMC3511845

